# The Postpharyngeal Gland: Specialized Organ for Lipid Nutrition in Leaf-Cutting Ants

**DOI:** 10.1371/journal.pone.0154891

**Published:** 2016-05-05

**Authors:** Pâmela Decio, Alexsandro Santana Vieira, Nathalia Baptista Dias, Mario Sergio Palma, Odair Correa Bueno

**Affiliations:** 1 Centro de Estudos de Insetos Sociais, Instituto de Biociências, Universidade Estadual Paulista, Rio Claro, São Paulo, Brazil; 2 Laboratório de Biologia Estrutural e Zooquímica, Centro de Estudos de Insetos Sociais, Instituto de Biociências, Universidade Estadual Paulista, Rio Claro, São Paulo, Brazil; CNRS, FRANCE

## Abstract

There are several hypotheses about the possible functions of the postpharyngeal gland (PPG) in ants. The proposed functions include roles as cephalic or gastric caeca and diverticulum of the digestive tract, mixing of hydrocarbons, nestmate recognition, feeding larvae, and the accumulation of lipids inside this gland, whose origin is contradictory. The current study aimed to investigate the functions of these glands by examining the protein expression profile of the PPGs of *Atta sexdens rubropilosa* (Hymenoptera, Formicidae). Mated females received lipid supplementation and their glands were extracted and analyzed using a proteomic approach. The protocol used combined two-dimensional electrophoresis and shotgun strategies, followed by mass spectrometry. We also detected lipid β-oxidation by immunofluorescent marking of acyl-CoA dehydrogenase. Supplying ants with lipids elicited responses in the glandular cells of the PPG; these included increased expression of proteins related to defense mechanisms and signal transduction and reorganization of the cytoskeleton due to cell expansion. In addition, some proteins in PPG were overexpressed, especially those involved in lipid and energy metabolism. Part of the lipids may be reduced, used for the synthesis of fatty alcohol, transported to the hemolymph, or may be used as substrate for the synthesis of acetyl-CoA, which is oxidized to form molecules that drive oxidative phosphorylation and produce energy for cellular metabolic processes. These findings suggest that this organ is specialized for lipid nutrition of adult leaf-cutting ants and characterized like a of diverticulum foregut, with the ability to absorb, store, metabolize, and mobilize lipids to the hemolymph. However, we do not rule out that the PPG may have other functions in other species of ants.

## Introduction

The salivary system of ants generally consists of four pairs of glands, namely, the salivary glands in the thorax, hypopharyngeal glands, mandibular glands [1], and postpharyngeal glands (PPGs). The PPG occurs mainly in ants and in some species of solitary wasps [2,3]. In ants, regardless of sex, caste, or lifestyle, these glands occur in pairs and are located dorsally in the transition between the pharynx and esophagus [4–6]. They are of ectodermal origin, formed during post-embryonic development from two dorsal evaginations of the pharyngeal epithelium [1,7].

There are several hypotheses about the possible functions of PPGs. Some authors [8,9] suggested that they are responsible for feeding larvae. It was proposed that the postpharyngeal glands act as diverticulum of the digestive tract [1,10]. Other researchers believed that these glands play the role of cephalic or gastric caeca [11–13]. Another hypothesis suggested that they mix exogenous and endogenous hydrocarbons, obtained during trophallaxis, creating a characteristic odor in the colony [14]. In addition, it was demonstrated that, in several ant species, PPGs contain the same long-chain hydrocarbons that are found in the cuticle [15]. PPGs are involved in recognition on the social level from individual to species, and hydrocarbons have been found to play important roles in olfactory communication [9]. However, these hydrocarbons might be taken in during grooming behavior [16].

While its origin is contradictory, the accumulation of lipid within PPGs has been observed in all species of ants. One possibility is that lipid results from secretions of the glandular epithelium itself. Another possibility is that it comes from food eaten. Three hypotheses concerning the lipid in PPG were proposed: 1) transcellular pathway—the material would enter the glandular cells and be metabolized; 2) extraoral route—the material would be regurgitated directly from the glands to other individuals of the colony; and 3) digestive route—the material would pass from the inside of the glands towards the crop and then into the ventricle [12]. Labeled isotope analysis revealed that molecules of fatty acids and triacylglycerides injected into the hemolymph did not reach the interior of the PPGs of *Solenopsis invicta* fire ants, suggesting that lipids would probably come from food [13]. According to Bueno [17], PPGs in *Atta sexdens rubropilosa* are capable of lipid selectivity possibly because of the presence of hair in the final region of the pharynx or by capillarity, wherein lipid compounds, initially separated from non-lipids, reach the lumen of PPG and cross the cuticle to the interior by diffusion. If the amount of lipid ingested is large and the system is unable to store it, the excess of lipid is transported to the crop without reaching the midgut. When there is reduced stock, lipid returns through the esophagus and reaches the lumen of the gland.

Other authors [18] believed that PPGs of *Camponotus pennsylvanicus* are responsible for the digestion of ingested fats since they present digestive enzymes in the lumen, mainly lipases. However, in enzymatic assays of the same gland in *Acromyrmex octospinosus*, low α-glucosidase and esterase activities were detected [19], suggesting that PPGs have no digestive function in these ants. Moreover, it was observed that the gland cells contained abundant smooth endoplasmic reticulum and lipid droplets, indicating that these cells represent or participate in the composition of the absorbed nutritional supplement [6].

Toxicological studies of *A*. *sexdens rubropilosa* indicated a possible alternative intake route for the active ingredient of ant baits containing soybean oil as a vehicle [20,21]. For the control of insect pests, such as leaf-cutting ants, mineral or vegetable oils have been used in many tests as pesticide additives or synergists [22]. According to Chapman [23], the use of oil in the control of these insects has some advantages. These organisms are less resistant to oils; they are not harmful to human health, and they are economically more viable and easier to be obtained than commercial insecticides. The use of oil in this experiment can show how this vegetable oil contributes to the nutrition of leaf-cutting ants.

The protein expression analysis provides important information such as expressed proteins, level and time of expression of these proteins, and proteins expressed in response to different situations or treatments [24,25]. Taking into account information about the presence of lipids within the PPG and recent reports on the increase in mitochondria and peroxisomes population in glandular cells of workers of *A*. *sexdens rubropilosa* ants supplemented with lipids compared with those non-supplemented with lipids [26]. Mitochondrial organelle is responsible for the β-oxidation of fatty acids, a process wherein fatty acids are converted to acetyl-CoA, which then enters the citric acid cycle to be further oxidized and generate ATP [27,28]. The immunofluorescent marking acyl-CoA dehydrogenase in the PPGs allows detection of β-oxidation of fatty acids.

Only a few works have attempted to identify the major enzymes in PPGs [18,19] and most of the proteomic studies of ants are restricted to the venoms produced by some species [29–31]. Because of the presence of lipids in the gland lumen and within its cells, the current study aimed to investigate the functions of these glands by examining the protein expression profile of the PPGs of *A*. *sexdens rubropilosa*. We used a protocol that combines two-dimensional electrophoresis (SDS-PAGE) and shotgun strategies, followed by mass spectrometry analyses (MALDI ToF-ToF and LCMS-IT-ToF) and immunofluorescence analysis of acyl-CoA dehydrogenase from ants subjected to lipid supplementation.

## Results

### Two-dimensional electrophoresis and mass spectrometry and MALDI ToF-ToF

**Fig 1:**
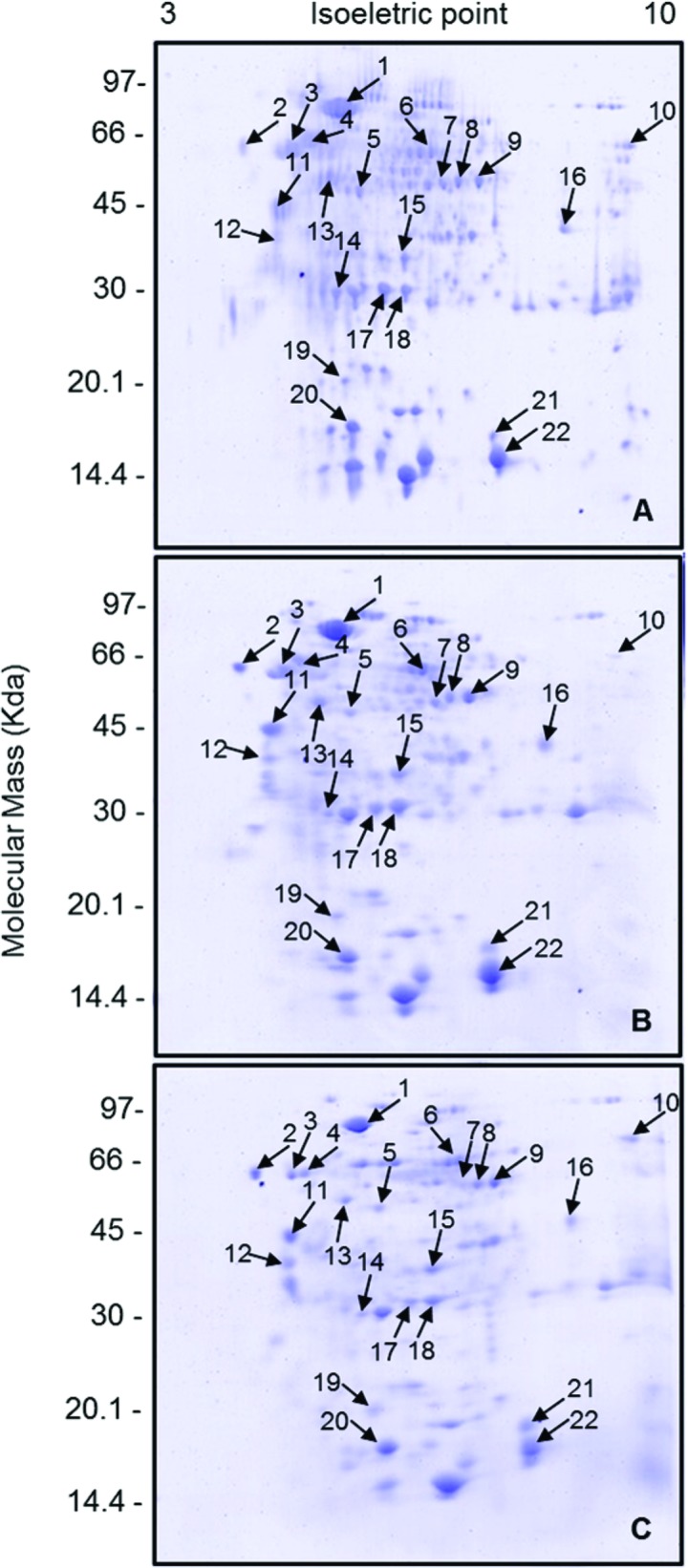
Protein profiles of postpharyngeal glands of mated females of *Atta sexdens rubropilosa*. (A) CONTROL group, without lipid supplementation. (B) LIPID 4 h group, dissected 4 h after lipid supplementation. (C) LIPID 120 h group, dissected 120 h after lipid supplementation. The numbered arrows indicate the protein spots identified. Profiles obtained on a 12.5% (w/v) SDS-PAGE gel stained with Coomassie Brilliant Blue R-250 (CBB).

The electrophoretic patterns of samples from three 2-DE gels were analyzed for each experimental condition. Image analysis revealed 166 protein spots in PPGs from the CONTROL group (Fig 1A) within the range of isoelectric point (pI) 3–9 and molecular mass (MM) of 16–94 kDa. In group LIPID 4 h (Fig 1B), 82 spots were observed (pI between 3–9 and MM: 16–99 kDa), while in group LIPID 120 h (Fig 1C) 89 spots were displayed (pI between 4–9 and MM: 17–135 kDa). From the three protein profiles obtained, the spots were cut and subjected to mass spectrometry (MALDI ToF-ToF). A total of 22 proteins were identified with the protein engine search MASCOT, with scores from 33 to 170 and sequence coverage from 1% to 16% (Fig 1 and Table 1).

**Table 1:** Proteins identified by MALDI ToF-ToF mass spectrometry.

Spot	Access code NCBI	Protein/reference	MM (kDa)/pI	Mascot Score	% coverage	MS/MS (ion score)
**1**	EFN66141	Fatty acyl-CoA reductase 1	78.9/5.0	49	2%	LEELTKNSVFNR (32)
**2**	EFN75351	Calreticulin	59.7/3.5	44	5%	NWVYSEHPGK (20)
						HEQNIDCGGGYVK (24)
**3**	EFN61064	E3 ubiquitin-protein ligase NRDP1	56.7/4.0	49	3%	TPITSAQLRAVPR (27)
**4**	EFN64123	Tubulin beta-1 chain	57.4/4.2	57	9%	GHYTEGAELVDSVLDVVR (19)
						LHFFMPGFAPLTSR (14)
						ISEQFTAMFR (24)
**5**	ABY55637	Arginine kinase	43.6/5.2	112	14%	LIDDHFLFK (21)
						FWPTGR (34)
						LPFSHNDR (57)
**6**	EFN61103	CKLF-like MARVEL transmembrane domain-containing protein 4	52.9/6.7	34	16%	AVLYMIAFIAQLSAWTAYR (34)
**7**	EFN63125	Glutamine synthetase 2 cytoplasmic	46.6/6.5	38	9%	NGFPGPQGPYYCGVGADK (29)
						RPSSNCDPYSVCDALVR (9)
**8**	EGI65281	Malate dehydrogenase	37.8/6.9	95	16%	AFNNIAAAFLVGAMPR (32)
						NVIIWGNHSSTQYPDAAHATVTLLSGLK (25)
						DIVFSFPVIIK (39)
**9**	EFN62401	Isocitrate dehydrogenase (NADP)	51.0/7.4	143	9%	NILGGTVFR (24)
						AFAHSSFQYALSR (69)
						KIWYEHR (17)
						ETSTNPIASIFAWTR (38)
**10**	XP_001604215	PREDICTED: similar to 1D-myo-inositol-trisphosphate 3-kinase	73.7/9.4	33	2%	RNSCLASPNPYYTK (39)
**11**	EFN62865	CLIP-associating protein	39.4/4.0	51	10%	AAGDLLPLAMSSDGE Oxidation (M) (35)
**12**	EGI66394	Annexin-B9	33.2/3.9	60	3%	GFGTDEQAVLDVLAHR (38)
**13**	ACX37099	Beta actin	46.6/4.7	72	14%	VAPEEHPVLLTEAPLNPK (11)
						SYELPDGQVITIGNER (20)
						DLYANTVLSGGTTMYPGIADR (41)
**14**	EFN78063	Glutathione S-transferase	28.4/5.5	88	2%	LIYFPITALAEPIR (49)
						KRPPAIFSPN (45)
**15**	EFN88384	Protein tyrosine phosphatase domain-containing protein 1	31.8/6.4	37	1%	GPSNLGGLWR (37)
**16**	EGI70407	Rhophilin-2	35.8/8.2	33	1%	LVTPMDR Oxidation (M) (33)
**17**	EFN76290	Putative aminopeptidase W07G4.4	26.0/5.6	63	3%	NSVGENCYVSDEVITAK (27)
**18**	EFN89160	Peroxiredoxin – 6	28.8/6.5	170	16%	LKDHVDWVNDIK (65)
						DHVDWVNDIK (23)
						VIDSLQLVDR (53)
						RPEIATPANWVPGEK (29)
**19**	Q1Z7T2_PHOPR	Putative long-chain fatty acid transport protein	18.9/5.0	45	2%	NPAAMAMFDR Oxidation (M) (39)
**20**	ADE27971	fatty acid binding protein	14.1/5.7	44	6%	VDDIVCTR (44)
**21**	EFN67743	Protein abnormal spindle	17.9/7.3	52	1%	INMYIVKGALCVR Oxidation (M) (34)
						MTMYSRK Oxidation (M) (14)
**22**	XP_003424621	PREDICTED: acid sphingomyelinase-like phosphodiesterase 3b-like	17.23/7.4	49	2%	RLMGLLLPALLLLAR (49)

Proteins identified from spots obtained by two-dimensional electrophoresis of extracts from postpharyngeal glands of *Atta sexdens rubropilosa* from groups CONTROL, LIPID 4 h, and LIPID 120 h. NCBI = National Center for Biotechnology Information. MM = Molecular Mass (kDa). pI = Isoelectric Point. % coverage = Percentage of sequence coverage identified. Mascot score: protein scores higher than 30 were considered significant.

**Fig 2:**
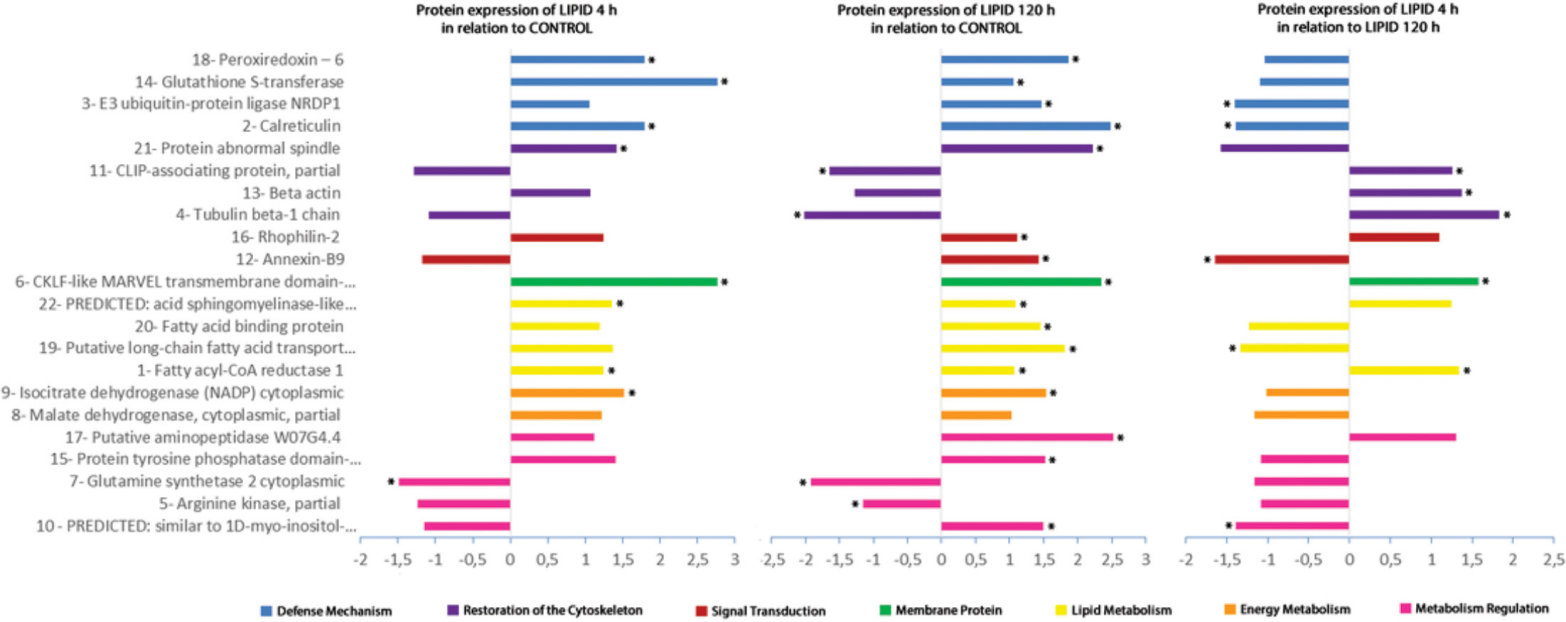
Differential expression of proteins identified from postpharyngeal glands of *Atta sexdens rubropilosa*. The x-axis indicates increase (overexpression) or decrease (underexpression) in spot volumes on gels in the comparisons between groups CONTROL and LIPID 4 h; CONTROL and LIPID 120 h; and LIPID 4 h and LIPID 120 h. Proteins represented by bars with * showed statistically significant differences in expression (p < 0.005).

The correspondence of spots for comparisons and differential expression analysis was performed using the algorithm ImageMaster 2-DE Platinum 7.0 for the gels that were representative of each experimental condition. According to statistics, spots that differed by 1.5 were differentially expressed (up- or down-regulated) between groups (Fig 2). The images obtained by two-dimensional electrophoresis were analyzed for the number of matches made with the proteins identified in the comparisons of the experimental groups. From the total number of matches obtained, there was a greater difference in protein expression between groups CONTROL and LIPID 120 h (90.9%) than in the other comparisons—CONTROL x LIPID 4 h (31.8%) and LIPID 4 h x LIPID 120 h (45.5%).

The proteins identified were divided into functional groups to facilitate the discussion. The proteins classified as belonging to the **Defense Mechanism** were peroxiredoxin 6 (spot 18), glutathione S-transferase (spot 14), E3 ubiquitin-protein ligase Nrdp1 (spot 3), and calreticulin (spot 2). The proteins involved in the process of **Restoration of the Cytoskeleton** were abnormal spindle protein (spot 21), CLIP-associating protein (spot 11), Beta-actin (spot 13), and Tubulin beta-1 chain (spot 4). Rhophilin-2 (spot 16) and Annexin-B9 (spot 12) were classified as proteins participating in **Signal Transduction** and only CKLF-like MARVEL transmembrane domain-containing protein 4 (spot 6) was identified as a **Membrane Protein**. The proteins related to **Lipid Metabolism** were predicted: acid sphingomyelinase-like phosphodiesterase 3b-like (spot 22), fatty acid binding protein (spot 20), putative long-chain fatty acid transport protein (spot 19), and fatty acyl-CoA reductase 1 (spot 1). The following proteins were identified as being associated with **Energy Metabolism**: cytoplasmic isocitrate dehydrogenase (NADP) (spot 9) and cytoplasmic malate dehydrogenase (spot 8). The proteins involved in **Metabolic Regulation** were putative aminopeptidase W07G4.4 (spot 17), domain-containing protein tyrosine phosphatase 1 (spot 15), glutamine synthetase 2 cytoplasmic (spot 7), arginine kinase (spot 5), and predicted: similar to 1D-myo-inositol-trisphosphate 3-kinase (spot 10). According to Fig 2, there is a general trend of overexpression in most of proteins identified in the presence of oil.

### Mass spectrometry—LCMS-IT-ToF

To increase the number of proteins identified in this study, experiments using shot-gun analysis were performed with the three groups of *A*. *sexdens rubropilosa* (CONTROL, LIPID 4 h, and LIPID 120 h). The data generated by the LCMS-IT-ToF mass spectrometer are qualitative in this case. Table 2 presents the list of proteins identified from this second technique used in the study.

**Table 2:** List of proteins identified by LCMS-IT-ToF mass spectrometry.

Experimental Group	Functional Group	Access code NCBI	Protein/reference	% Cobertura	Mascot Score	Sequência (Ion Score)
Control	Defense Mechanism	XP_624109	PREDICTED: ubiquitin-like protein 4A-like isoform 2	4%	32	KVIVKK (32)
Control	Defense Mechanism	EGI70251	Protein disulfide-isomerase	4%	50	YKPDKPEITTENVLEFVTAFVEGK (50)
Control	Energy Metabolism	AAG17159	Cytochrome c oxidase subunit II	3%	20	LLDTANR (20)
LIPID 4 h	Defense Mechanism	EGI70566	Glutathione S-transferase	2%	84	LIYFPITALAEPIR (47)
						KIDQSTAISR (17)
						IDQSTAISR (20)
LIPID 4 h	Defense Mechanism	EGI69239	Transferrin	5%	54	DRYDCIER (13)
						TKEEPDAIYR (19)
LIPID 4 h	Defense Mechanism	EGI70251	Protein disulfide-isomerase	4%	22	YKPDKPEITTENVLEFVTAFVEGK (22)
LIPID 4 h	Defense Mechanism	EGI69239	Transferrin	5%	54	DRYDCIER (13)
						TKEEPDAIYR (19)
LIPID 4 h	Defense Mechanism	EGI70251	Protein disulfide-isomerase	4%	22	YKPDKPEITTENVLEFVTAFVEGK (22)
LIPID 4 h	Metabolic Regulation	EFN69158	Glyceraldehyde-3-phosphate dehydrogenase	10%	50	VVHDNFEIVEGLMTTVHAITATQK (44)
LIPID 4 h	Signal Transduction	EGI69223	Tyrosine-protein phosphatase non-receptor type 9	4%	38	ILTLLK (30)
LIPID 4 h	Lipid Metabolism	EGI69897	Putative fatty acyl-CoA reductase	3%	23	LYQLNENDSMLKDSRR (23)
LIPID 4 h	Energy Metabolism	AAF69222	Cytochrome c oxidase subunit II	2%	22	LINNK (22)
LIPID 120 h	Defense Mechanism	EGI69034	Peroxidasin-like protein	2%	31	NLPMASPK (24)
LIPID 120 h	Restoration of cytoskeleton	ACX37099	Beta actin	5%	23	AVFPSIVGRPR (19)

List of proteins identified from extracts of postpharyngeal glands of *Atta sexdens rubropilosa* from groups CONTROL, LIPID 4 h, and LIPID 120 h. NCBI = National Center for Biotechnology Information. % coverage = Percentage of sequence coverage identified. Mascot score: protein scores higher than 20 were considered significant.

### β-Oxidation of fatty acids

**Fig 3:**
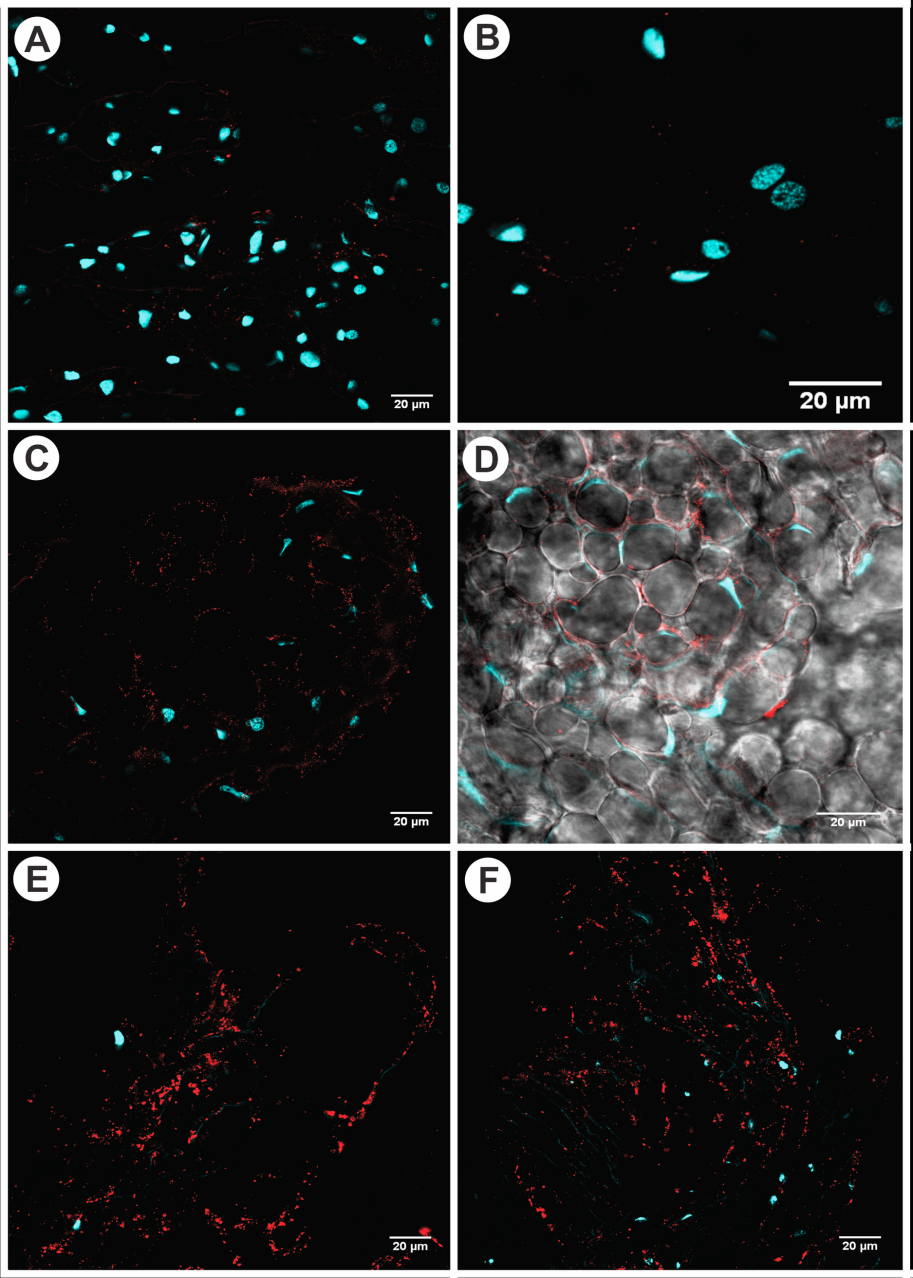
Confocal microscopy of postpharyngeal glands of *Atta sexdens rubropilosa* showing the fluorescence of the enzyme acyl-CoA dehydrogenase. (A-B)—Detail of the digitiform prolongation showing the nuclei of glandular cells of mated females from the group without lipid supplementation (FAST 120 h), stained blue (DAPI), and the low enzymatic activity of acyl-CoA dehydrogenase (red) in the glandular cells. (C-D)—Detail of the digitiform prolongation showing the nuclei of glandular cells in the PPG of mated females from the group supplemented with lipids (LIPID 48 h), stained blue (DAPI), and the moderate enzymatic activity of acyl-CoA dehydrogenase (red) in the glandular cells. It can be seen that the enzyme acyl-CoA dehydrogenase is primarily distributed along the lateral area of glandular cells (DIC) of the digitiform prolongation in the PPG. (E-F)—Detail of the digitiform prolongation showing the nuclei (blue) of glandular cells in the PPG of mated females from the group supplemented with lipids (LIPID 120 h), stained blue (DAPI), and the high enzymatic activity of acyl-CoA dehydrogenase (red) in the glandular cells.

Indirect immunofluorescence analysis of the PPGs of *A*. *sexdens rubropilosa* mated females revealed weak fluorescence emission (red), suggesting low enzymatic activity of acyl-CoA dehydrogenase in glandular cells of PPGs in group FAST 120 h (Fig 3A and 3B). Moderate fluorescence intensity was observed, indicating moderate enzymatic activity of acyl-CoA dehydrogenase in glandular cells of the PPGs in group LIPID 48 h (Fig 3C and 3D). Moreover, strong fluorescence intensity was noticed, signifying high enzymatic activity of acyl-CoA dehydrogenase in glandular cells of the PPGs in group LIPID 120 h (Fig 3E and 3F). It can be seen that acyl-CoA dehydrogenase is primarily distributed along the lateral area of glandular cells of the finger-like projections in the PPG (Fig 3C and 3D).

Fig 3D shows the maximum projection image of the fluorescence emission, in red, by acyl-CoA dehydrogenase within the glandular cells of the postpharyngeal gland (DIC), as well as the compressed nuclei of these same cells (Fig 3C) for group LIPID 48 h (Fig 3A) in relation to group FAST 120 h.

**Fig 4:**
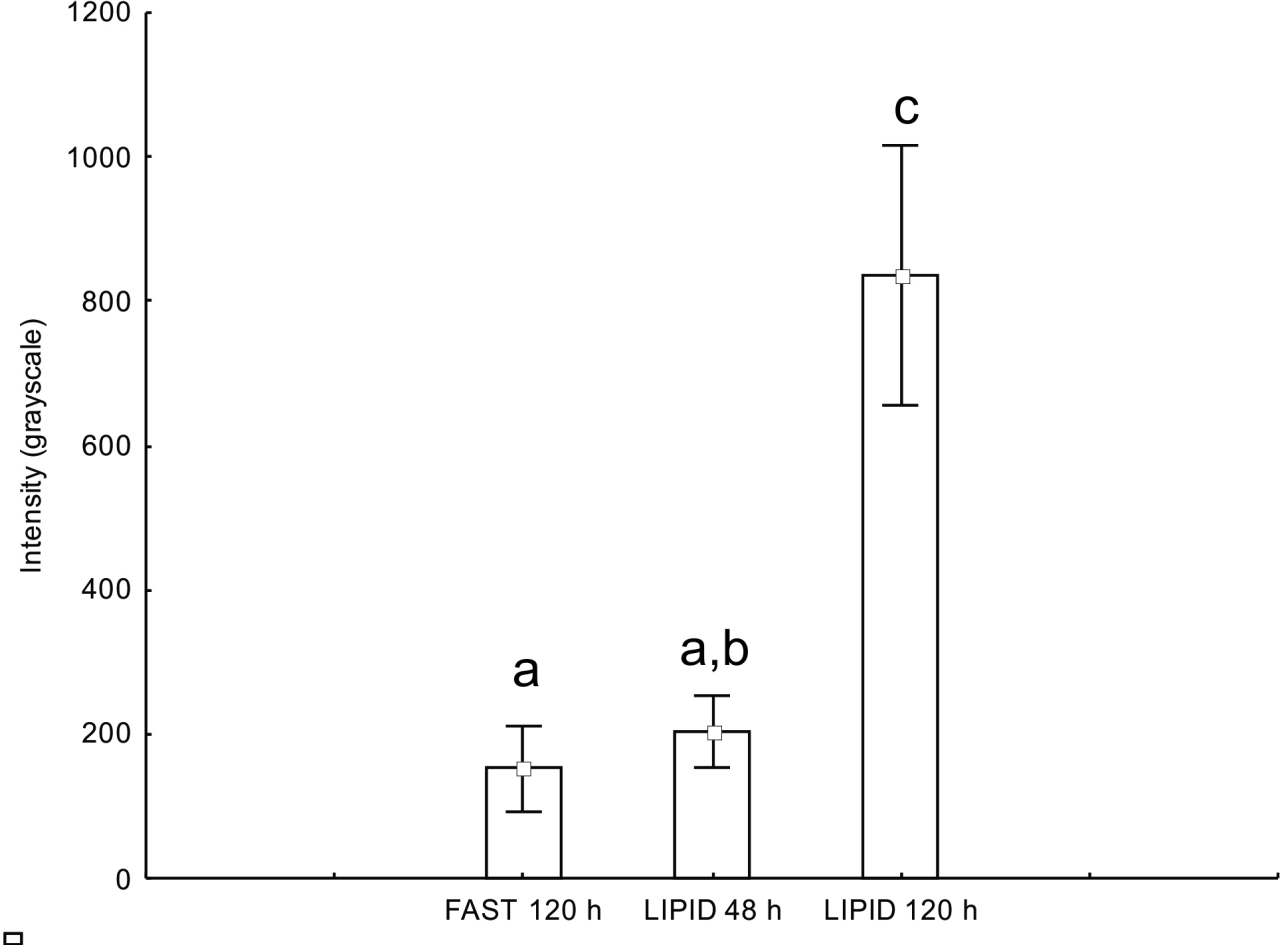
One-way ANOVA analyses. Intensity of emitted fluorescence (values in grayscale) in the measured areas of gland cells in the postpharyngeal glands of *Atta sexdens rubropilosa* mated females for the enzyme acyl-CoA dehydrogenase. Equal letters: there is no statistically significant difference (p > 0.05); different letters: there are significant statistical differences (p < 0.05).

Using the software ImageJ, the fluorescence emission intensity of the enzyme acyl-CoA dehydrogenase was measured. The parametric statistical test ANOVA revealed a significant difference between group FAST and group LIPID 48 h and LIPID 120 h (F = 86.7935, p < 0.0001). The posteriori Tukey test revealed that there was no significant difference between group FAST 120 h and group LIPID 48 h (p > 0.05), but there is between groups FAST 120 h and LIPID 120 h (p < 0.01), and groups LIPID 48 h and LIPID 120 h (p < 0.01) (Fig 4).

## Discussion

The analysis of the presence of lipids within the PPGs and the possibility of explaining this phenomenon have advanced our understanding of the metabolic functions of this gland. Overall, the results showed that the supply of lipids to newly mated females of *A*. *sexdens rubropilosa* causes a larger difference in protein expression in the PPGs (Fig 2), indicating that the lipid diet alter the proteins involved in cellular metabolism.

The main protein functional groups with and without lipid supplementation to the PPG were defense mechanism, restoration of the cytoskeleton, signal transduction, membrane protein, lipid metabolism, energy metabolism, and metabolic regulation. Regarding the defense mechanism, which showed the greatest difference in protein expression after lipid supplementation (LIPID 120 h), glutathione S-transferase protein (spot 14) and peroxiredoxin 6 (spot 18) stood out (Fig 2). Glutathione S-transferase is known to have a central role in biotransformation and elimination of xenobiotics [32]. On the other hand, peroxiredoxin 6 is a multifunctional peroxidase which has a protective function in an environment subjected to oxidative stress [33]. Peroxidasin (LCMS-IT-ToF; Table 2) also has a domain that functions as peroxidase [34]. Previous studies conducted by Falco [35] and Caetano [6] showed a large number of peroxisomes in the cytosol of cells of the PPG. It was also shown that there is an increase in the population of peroxisomes in cells of PPGs subjected to lipid supplementation [26]. Therefore, the supply of lipids to glandular cells of the PPG promotes overexpression of enzymes involved in cellular detoxification, possibly generating peroxides as products of this enzymatic action.

The cytoskeleton is responsible for providing structure and support to cells. They also play a role in intracellular transport, contractility, mobility, and reorganization of cells [27,36]. In this study, we observed some proteins involved in the restoration of the cytoskeleton. One of them was abnormal spindle protein (spot 21), which is important for the orientation of the mitotic spindle [37,38]. This protein showed overexpression in groups LIPID 4 h and LIPID 120 h, compared to CONTROL group (Fig 2). Two other proteins, Beta-actin (spot 13) and Tubulin beta-1 chain (spot 4), presented overexpression in group LIPID 4 h in relation to group LIPID 120 h (Fig 2). This suggests that intracellular lipids cause changes in the cytoskeleton from the first 4 h up to 120 h, indicating that these cells are capable of restoring the cytoskeleton in the presence of lipids due to cell expansion.

Furthermore, this study identified three proteins that play a role in signal transduction and are involved in the reorganization of the cytoskeleton. Annexin (spot 12) modulates cytoskeleton organization [39], while Rophilin-2 (spot 16) acts in signal transduction through the formation of complexes with other signaling molecules, playing an important role in cytoskeleton organization as well [40]. Both were overexpressed in group LIPID 120 h when compared to CONTROL group (Fig 2), and the same happened to tyrosine phosphatase (LCMS-IT-ToF; Table 2) found in group LIPID 4 h. This supports the aforementioned data, which indicate that, in the presence of lipid, there is increased cell signaling directly, involved in the reorganization of the cytoskeleton.

Fats are a highly efficient method of storing metabolic energy [28]. The simplest lipids derived from fatty acids are triacylglycerols, which are stored in the fat body of insects as lipid droplets [41]. Previous studies conducted reported the accumulation of lipids in the PPG [9,12,13,17,26,42]. In this study, analysis of lipid metabolism allowed identification of four differentially expressed proteins: fatty acyl-CoA reductase 1 (spot 1), putative long-chain fatty acid transport protein (spot 19), fatty acid binding protein (spot 20), and acid sphingomyelinase-like phosphodiesterase 3b-like (spot 22). Fatty acyl-CoA reductase 1 presented overexpression in the groups supplemented with lipids compared to CONTROL group (Fig 2). This is an enzyme of the oxidoreductase family, which catalyzes the formation of a fatty alcohol from an acyl-CoA [43] or exhibits long-chain-fatty-acyl-CoA reductase activity. Primary long-chain fatty alcohols are commonly found in their free form or as a component of wax esters in plants, insects, and mammals [43,44]. In enzyme tests with the PPG of *Acromyrmex octospinosus* [19], was detected low activity of esterases and α-glucosidase were detected. However, the detection of fatty acyl-CoA reductase indicates that the PPG is involved in the synthesis of fatty alcohols and that there is occurring reduction of this oil from lipid nutrition.

Putative long-chain fatty acid transport protein and fatty acid binding protein showed overexpression in group LIPID 120 h compared to CONTROL (Fig 2). These proteins act as carriers and direct the displacement of lipids into or out of the cell [45,46]. According to Vieira [47], the glandular cells of the PPG of *A*. *sexdens rubropilosa* would first play the role of storage and later of lipid mobilization to the hemolymph. Thus, we showed that increasing the lipid supply also promoted increased mobilization of lipids in the glandular cells into the hemolymph, and these two proteins (long-chain fatty acid transport and fatty acid binding) are responsible for lipid transport in glandular cells.

Another protein that was overexpressed when there was lipid supplementation was acid sphingomyelinase-like phosphodiesterase 3b-like (Fig 2). This is a hydrolase enzyme involved in the metabolic reactions of sphingolipids [48]. Sphingolipids constitute a class of lipids defined by their eighteen carbon amino-alcohol backbones and those play significant roles in membrane biology and provide many bioactive metabolites that regulate cell function.

In addition, in relation to lipid metabolism, a study [26] showed an increased population of mitochondria in glandular cells of the PPG of *A*. *sexdens rubropilosa*. Here, we investigated β-oxidation of these lipids by immunofluorescence, which revealed low activity of acyl-CoA dehydrogenase in the group without lipid supplementation (FAST 120 h). Furthermore, the same test revealed moderate to high activity of this enzyme in the group that received lipid supplementation (LIPID 48 and 120 h), as confirmed by ANOVA. In the β-oxidation process, fatty acids are converted into acetyl-CoA, one of the enzymes called acyl-CoA dehydrogenase, and go through the citric acid cycle to be further oxidized and generate ATP [27,28]. For example, the complete degradation of a fatty acid molecule with 16 carbon atoms releases 8 acetyl-CoA, 7 NADH, and 7 FADH2 during β-oxidation, and 24 NADH, 8 FADH2, and 8 GTP in the citric acid cycle, producing a total of 131 molecules of ATP after the respiratory chain [28]. Therefore, the glandular cells of the PPGs of mated females of *A*. *sexdens rubropilosa* would be using part of the lipids from the lipid supplementation for the synthesis of acetyl-CoA through β-oxidation of fatty acids. The molecules of acetyl-CoA can then be oxidized in the citric acid cycle to generate energy for all metabolic processes that occurs into glandular cells of PPG.

Consequently, two proteins identified take part in energy metabolism. Malate dehydrogenase (spot 8), which catalyzes the oxidation of L-malate to oxaloacetate [28], showed no significant difference in expression in any of the comparisons (Fig 2). However, the cytoplasmic enzyme isocitrate dehydrogenase (NADP) (spot 9), responsible for catalyzing the oxidative decarboxylation of citrate to form α-ketoglutarate [28], was overexpressed in both groups with lipid supplementation compared to the control (Fig 2). The Citric Acid Cycle, which is responsible for the oxidation of acetyl-CoA, produces two molecules of CO_2_ through the oxidation of isocitrate and α-ketoglutarate for each turn of the cycle. Although the pathway directly produces only one ATP per round, the four stages of the oxidation cycle supply the respiratory chain with a large flux of electrons from NADH and FADH_2_, which leads to the formation of a large number of molecules of ATP during oxidative phosphorylation [28,48]. Thus, the overexpression of isocitrate dehydrogenase indicates that, in the presence of lipids, there was an increase in NADH production, leading to an increase of ATP production in oxidative phosphorylation. We verified the presence of the enzyme cytochrome c oxidase subunit II (LCMS-IT-ToF; Table 2), which is the last protein of the electron transport chain and catalyzes the reduction of oxygen to water. In this process, four protons are translocated, which generates a chemiosmotic potential used by the ATP synthase for the production of ATP [28,48]. However, we suggest that part of the lipids may be used as a substrate for oxidative phosphorylation, generating energy for the glandular cells.

The research also revealed some proteins involved in metabolic regulation. The proteins 1D-myo-inositol-trisphosphate 3-kinase (spot 10) and tyrosine phosphatase domain-containing protein 1 (spot 15) showed increased protein expression when the ant group received lipid supplementation (Fig 2). Protein kinases are enzymes responsible for catalyzing phosphorylation, while phosphatases act in the process of dephosphorylation of the substrates [28,48]. Hence, glandular cells regulate cell metabolism in the presence of excess lipids.

According to Bueno (17), the functions of the PPGs are conflicting. Delage-Darchen (12) and Eelen et al. (9) suggested that PPGs are not involved in larval feeding. Histologically, this gland has no muscle tissue to make regurgitation for trophallaxis. They do not play the role of cephalic or gastric caeca responsible for the digestion of fats either [7,12,13,18,49,50] because of the low activity of digestive enzymes such as esterases and α-glucosidases [51] and gastric caeca occurs only in gut [23]. The PPGs would have the function of mixing exogenous and endogenous hydrocarbons obtained during trophallaxis [14], and are involved in recognition on the social level from individual to species (nestmate recognition) [9], but states that long-chain hydrocarbons found in the PPG could be taken in during grooming behavior [16]. Another relevant hypothesis is that in mated queens the gland probably plays a role in providing the queen with nutritious oils for egg production [9].The most plausible hypothesis in the literature was described by Delage-Darchen [12], which suggested that PPG is a diverticulum of the digestive tract and that its function would be related to nutrition. Here, the results allow us to affirm that PPG is a diverticulum of the foregut with ability for lipid nutrition of leaf-cutting ants, however, these glands have several functions that can differ among species.

Finally, this gland is involved in lipid and energy metabolism due to the increase of proteins expressed. From the data obtained, we infer that part of the lipids may either be reduced, used for the synthesis of fatty alcohol (fatty acyl-CoA reductase 1), transported to the hemolymph (putative long-chain fatty acid transport protein and fatty acid binding protein), or used as a substrate for the synthesis of acetyl-CoA (Acyl-CoA dehydrogenase), which is oxidized (malate dehydrogenase, cytoplasmic and isocitrate dehydrogenase [NADP] cytoplasmic) and forms molecules that contribute to oxidative phosphorylation (cytochrome c oxidase subunit II), to generate energy for cellular metabolic processes. It is concluded that this organ is specialized for lipid nutrition of adult leaf-cutting ants characterized like a diverticulum foregut, with the ability to absorb, store, metabolize, and mobilize lipids to the hemolymph. Furthermore, we do not rule out that PPG has other functions in other species of ants.

## Materials and Methods

For the experiments, newly mated females were used because, before their nuptial flight [52], winged females of *A*. *sexdens rubropilosa* double their corporal mass with lipids and energetic content that are used for the flight as for excavation and foundation of the nest [53].

Newly mated females collected in the field were used for the extraction of the PPGs. The collections were carried out during the swarms of October-November 2010, 2011, and 2013 (Campus of the University of the State of São Paulo, Rio Claro, SP, Brazil, not necessary collection permission). Soon after nuptial flight, the female *A*. *sexdens rubropilosa* returned to the ground and lost their wings, they were collected and placed individually into plastic containers (200 mL) lined with moistened plaster and capped. The containers were then transferred to the laboratory for further analysis.

### Bioassay for proteomic analysis

A total of 300 newly mated females were used for each protein identification technique, divided into three groups of 100 individuals: CONTROL, LIPID 4 h, and LIPID 120 h. In the first group, there was no food supplementation, whereas newly mated females of the second and third groups received 2 μL of triacylglycerol—lipid (soybean oil) in the mouthparts and fasted for 4 h and 120 h, respectively, after lipid supplementation. The amount of lipid and dissection times were defined according to Bueno [16]. The females were kept in a biological oxygen demand (BOD) incubator at 25 ± 1°C and a relative humidity of 70%.

The newly mated females were anesthetized by cooling at 4°C, their heads were removed, and then the PPGs were dissected in Milli-Q water. With the aid of entomological forceps, the head was opened and the glands were then removed. Once dissected, the glands were immediately stored in siliconized microtubes (1.5 mL) with the protease inhibitor phenylmethylsulfonyl fluoride (PMSF) and kept at -80°C until used.

### MALDI ToF-ToF experiment

#### Protein assay

For protein extraction, the material was washed in a solution containing 1 mM PMSF, macerated, and centrifuged at 8000 rpm for 15 min. Due to the high amount of lipid, 0.1% sodium dodecyl sulfate (SDS) was added for 35 min in the three groups of mated females to solubilize lipids associated with the protein sample. The 2D Clean-Up Kit (GE Healthcare, Sweden) was also used to avoid the presence of impurities or nuisance compounds during isoelectric focusing and two-dimensional electrophoresis. Protein quantification was determined by the Bradford method [54] using bovine serum albumin (BSA) as a standard.

#### Two-dimensional gel electrophoresis

Immobiline DryStrips, linear pH 3–10, 7 cm long (Amersham Biosciences, Uppsala, Sweden) were rehydrated overnight at 25°C with 250 μg of protein in 125 μL of rehydration solution (8 M urea, 2% CHAPS, 0.2% DTT, 0.5% IPG Buffer pH 3–10, 0.002% bromophenol blue) for each treatment. After rehydration, the first-dimension isoelectric focusing (IEF) was performed using the IPGphor system (GE Healthcare). The proteins were separated at 20°C using 50 μA per strip with the following linear voltage increase: 200 V for 2 h, 1000 V for 1 h, and 5000 V for 1.5 h. The strips were then incubated in equilibration buffer [50 mM Tris-HCl, pH 8.8, 6 M urea, 30% (v/v) glycerol, 2% (w/v) SDS] containing 0.5% (w/v) dithiothreitol (DTT) for 15 min, followed by equilibration buffer containing 4% (w/v) iodoacetamide (IAA) for 15 min.

The IPG strip was then transferred onto SDS-PAGE [12.5% (w/v) polyacrylamide and 0.8% (w/v) bis-(N,N’-methylenebisacrylamide)] and the second dimension was performed at 10 mA/gel for 15 min and 20 mA/gel for 1 h at 10°C in a Mini-VE system (GE Healthcare). The gels were stained overnight with Coomassie Brilliant Blue R-250 (CBB) and stored at 21°C in preserving solution [7% (v/v) acetic acid].

#### Image acquisition, protein quantitation, and statistical analysis

Three gels were obtained from each treatment and control group, 33 of the 100 individuals of a group were used for one replicate. The gels were scanned and digitized (BioImage, GE Healthcare) in the transparency mode at 300-dpi resolution for documentation. All identified proteins were subjected to quantitative expression analysis.

All images were analyzed by the ImageMaster 2D Platinum 7.0 software (GE Healthcare, Sweden). The gel with the most spots was chosen as a reference and used to match the corresponding protein spots between gels. Paired comparisons were made between all groups (CONTROL and LIPID 4 h; CONTROL and LIPID 120 h; LIPID 4 h and LIPID 120 h). The parameters used for normalization, spot matching, and expression analysis were as described in the user guide and further manual editing was performed to correct mismatched and unmatched spots. A probability of p<0.005 was considered to be statistically significant according to ANOVA.

#### In-gel digestion

Protein spots which showed significant changes after treatment were excised from replicated gels. Each excised protein spot was destained with 50 mM ammonium bicarbonate / 50% acetonitrile (MeCN) twice for 30 min, dehydrated in the presence of MeCN 100% at 25°C, and dried in a speed-vac system for another 30 min. Thereafter, the gel pieces were treated with 10 mM DTT in 50 mM ammonium bicarbonate for 1 h at 56°C followed by treatment with 55 mM IAA in 50 mM ammonium bicarbonate for 45 min in the dark at 25°C. Then, the gel pieces were washed with 25 mM ammonium bicarbonate and MeCN and dried in a speed-vac again. The spots were treated with 25 μL of trypsin solution (20 μg/mL, Promega, Madison, USA) in 50 mM ammonium bicarbonate, pH 7.9, at 37°C for 16 h. A solution of 50% acetonitrile (v/v) and 5% formic acid (v/v) was then added to obtain tryptic digests. The supernatants were collected, dried, and then kept at -80°C until the mass spectrometry analysis.

#### Mass spectrometry analysis and MALDI ToF-ToF

Samples were resuspended by adding 5 μL of 50% MeCN solution (v/v) [containing trifluoroacetic acid (TFA) 0.5% (v/v)]. A volume of 0.5 μL of each sample containing the tryptic digests was deposited on a MALDI plate. In addition, 0.5 μL of matrix [10 mg/mL a-cyano-4-hydroxycinnamic acid (CHCA) in methanol/acetonitrile (1:1, v/v)] was added to the sample, which was left to dry at 25°C for the mass spectrometric experiments. The analysis was performed with a matrix-assisted laser desorption/ionization time-of-flight/time-of-flight mass spectrometry (MALDI ToF/ToF–MS) instrument (Shimadzu, Axima Performance). The MS and MS/MS spectra were acquired in the positive-ion reflectron mode using an N_2_ laser, with monoisotopic peaks in the range between m/z 800 and 3500. For the MS/MS experiments, at least the five most intense ions from each MS spectrum were selected.

After all data acquisition, they were exported to be submitted to automatic analysis using the bioinformatics tool MASCOT Ion Search version 2.0 for access to databases.

### LCMS-IT-ToF experiments

#### In-solution digestion for shotgun strategy

A sample of 500 μg of protein was extracted from each experimental group and solubilized in 50 μL of 50 mM ammonium bicarbonate (pH 7.9) containing 7.5 M urea for 1 h at 37°C. Samples were reduced with 10 mM DTT at 37°C for 1 h and alkylated with 40 mM IAA at 25°C for 1 h in the absence of light. All samples were then diluted five times with 100 mM ammonium bicarbonate, pH 7.8, and calcium chloride at a concentration of 1 mM. Trypsin (Promega) was added to the denatured protein solution (1:50 w/w trypsin:protein) for 18 h at 37°C. The reaction was stopped with 5 μL of formic acid. The digested samples were desalted using a 1 cc tC18 Sep-Pak Vac cartridge container (WATERS) and the tryptic peptides were solubilized in 50% MeCN for mass spectrometry analysis.

#### Mass spectrometry analysis and LCMS IT-ToF

The samples were analyzed using an LCMS-IT-ToF system. The system was equipped with an XBrigde™ BEH300 C18 column, 3.5 μm, 2.1 mm × 100 mm (Waters), 120-Å pore size, at 40°C. An optimized sample amount used in all analyses was adjusted to equal 500 μg of the extract. The mobile phase, delivered at 0.2 mL/min, consisted of bi-distilled water containing: (i) 0.05% (v/v) trifluoroacetic acid (solvent A) and (ii) 95% (v/v) acetonitrile in water containing 0.05% (v/v) trifluoroacetic acid (solvent B). The 90 min elution was performed using a gradient from 5% to 95% of solvent B and was monitored by light absorption at 214 nm.

MS and MS^n^ analyses were conducted on an ion trap/time-of-flight mass spectrometer (IT-ToF/MS) (Shimadzu, Kyoto, Japan) equipped with an electrospray ionization source. The instrument was set to permit the accumulation of all ions in the octapole, followed by rapid pulsing into the IT for MS^n^ analysis, and then introduction into the ToF sector for accurate mass determinations. The setting conditions for optimized operations were: positive mode, electrospray voltage 4.5 kV, CDL temperature 200°C, block heater temperature 200°C, nebulizer gas (N_2_) flow of 1.5 L/min, trap cooling gas (Ar) flow of 95 mL/min, ion trap pressure 1.7×10^−2^ Pa, ToF region pressure 1.5×10^−4^ Pa, ion accumulation time 50 ms, collision energy set at 50% both for MS_2_ and MS_3_, and collision gas set to 20%. The auto-tuning was performed with a Na-TFA solution and showed the following parameters: for the positive mode, error 3.08 ppm, and resolution 15,000. The spectra obtained were first analyzed with the software tool LCMS solution (SHIMADZU), used to control data acquisition and analysis.

### Protein identification

MASCOT searches were conducted using MASCOT 2.2.06 (Matrix Science, London, UK) against the latest NCBI database (http://blast.ncbi.nlm.nih.gov, on October 3th, 2015) initially restricted to the taxon Hymenoptera (863,152 entries). Unidentified proteins were then subjected to new searches using the taxa Formicidae, among other gene banks for ant species such as *Camponotus floridanus*, *Harpegnathos saltator*, *Atta cephalotes*, *Solenopsis* spp., *Acromyrmex* spp., and *Pachycondyla* spp. (with a total of 152.017 entries); the proteins not identified were then searched using the taxa Arthropoda (3,642,270 entries) and Metazoa (15,023,936 entries). The search parameters were set as follows: peptide mass tolerance of 0.5 Da, a fragment mass tolerance of 0.8 Das, and the number of missing cleavages equal to 1. The modifications of peptides considered were: carbamidomethylation and oxidation methionine. The values of “ion score” and “protein score” considered significant were the pre-set values of the bioinformatics tool used. Proteins were considered identified when at least two peptides were assigned to the respective sequence. The proteins identified after the database search were subjected to additional filtering using false discovery rate (FDR) of less than 1%. FDR was calculated from forward and decoy matches by requiring significant matches for at least 2 distinct sequences. According to a Local FDR algorithm implemented in Scaffold, the peptide probability was set to a minimum of 90%, whereas the protein probability was set at 95%. The databanks mentioned above were appended with common external contaminants from cRAP, a maintained list of contaminants, laboratory proteins, and protein standards provided through the Global Proteome Machine Organization

### Bioassay for β-oxidation of fatty acids

Complementary to two-dimensional electrophoresis (10^−12^), fluorescence marking (10^−15^), which is more sensitive for the detection of acyl-CoA dehydrogenase was performed. In this bioassay, after 30 days of the nuptial flight, 24 mated females were used and divided into 3 groups: group without lipid supplementation and fasted for 120 h (FAST 120 h) and groups supplemented with 2 μL of TAG and fasted for 48 and 120 h (LIPID 48 h and LIPID 120 h, respectively). The ants were placed individually into plastic containers (200 mL) and kept in a biochemistry oxygen demand (B.O.D.) incubator at 25 ± 1°C and a relative humidity of 70%.

The mated females were anesthetized by cooling at 4°C and the PPGs were extracted in insect saline solution (0.128 M NaCl, 0.016 M Na_2_HPO_4_, and 0.019 M KH_2_PO_2_, pH = 7.2) for analysis in confocal microscopy.

### Confocal Laser Scanning Microscopy

#### Indirect immunofluorescence to detect β-oxidation of fatty acids

The 24 PPGs were fixed in 4% paraformaldehyde for 30 min and then permeabilized with 0.1% Triton® X-100 for 30 min. The PPGs were incubated overnight at 4°C with 10 μg/mL of anti-acyl-CoA dehydrogenase primary mouse monoclonal antibody (Very long-chain acyl-CoA dehydrogenase, VLCAD, monoclonal antibody, Molecular Probes). Additionally, Cy5-conjugated goat anti-mouse IgG (H+L) (Molecular Probes) diluted 1:1,000 for 1 h was used as secondary antibody. A solution of 10% goat serum was used as a blocking agent at all stages. The cell nucleus was counterstained with DAPI.

The markings (VLCAD and nucleus) in the PPGs were documented in full assembly by making use of a confocal laser scanning microscope (Zeiss LSM780-NLO). Fluorescent images were obtained using lasers with 405 nm and 633 nm excitation wavelengths. The optical sections were acquired in suitable Z-axis sectioning step sizes (0.63 μm and 0.67 μm). Different modules of the software Zeiss LSM780-NLO, as well as the software ImageJ, were used for the confocal analysis, including maximum projection.

In order to verify possible fluorescence emissions, PPG incubated only with Cy5 secondary antibody was used as the negative control.

#### Statistical analysis of the fluorescence emissions

The intensity of the fluorescence emitted (grayscale) by the enzyme acyl-CoA dehydrogenase was quantified using the ImageJ software. The Shapiro-Wilk test was carried out to assess normal distribution of data using the statistical software BioEstat, and then the parametric statistical test one-way ANOVA was performed, a pairwise Tukey test was used to check which groups were different from each other, using the software Statistica 7.
